# PD190 The Brazilian Health Technology Assessment Network Strategy: Capacity Building And Its Importance For The Sustainability Of The Public Health System

**DOI:** 10.1017/S0266462324004124

**Published:** 2025-01-07

**Authors:** Barbara Pozzi Ottavio, Marcela Freitas, Lais Barbosa, Maria Stella D’agostini, Rafaella Nóbrega, Ávila Vidal, Luciene Bonan

## Abstract

**Introduction:**

The Brazilian Health Technology Assessment (HTA) Network (REBRATS) dates to 2008, when the Ministry of Health (MoH) launched a call to register Brazil’s first HTA groups to promote and disseminate HTA in the country. To understand whether this strategy is succeeding, this paper evaluated the actions of REBRATS and the degree of advancement of the HTA field in Brazil.

**Methods:**

The following data on the composition of REBRATS were collected and analyzed: the number of HTA groups and professionals registered in the network, the number of professionals that have benefited from qualifying courses, and the evolution of the Brazilian National Committee for Health Technology Incorporation (CONITEC).

**Results:**

REBRATS expanded from 24 HTA groups to 112 groups in 2023, which includes over 800 professionals. From 2019 to 2023, the MoH financed over 90 courses, which have benefited more than 1,000 professionals. More HTA groups (from five to 23) have been hired to support CONITEC, enabling it to respond to a higher demand and more complex topics. These groups have been contributing to the development of HTA by assisting the MoH in defining a cost-effectiveness threshold; evolving the assessment of medical devices and new technologies (i.e., gene and targeted therapies); and creating methodological guideline–there will be 21 by 2024.

**Conclusions:**

Although the composition and technical capability of its member groups vary greatly, REBRATS has consolidated itself as a key strategy to support decision-making regarding technologies.

